# Systematic Identification of Core Transcription Factors Mediating Dysregulated Links Bridging Inflammatory Bowel Diseases and Colorectal Cancer

**DOI:** 10.1371/journal.pone.0083495

**Published:** 2013-12-26

**Authors:** Yun Xiao, Huihui Fan, Yunpeng Zhang, Wenjing Xing, Yanyan Ping, Hongying Zhao, Chaohan Xu, Yiqun Li, Li Wang, Feng Li, Jing Hu, Teng Huang, Yanling Lv, Huan Ren, Xia Li

**Affiliations:** 1 College of Bioinformatics Science and Technology, Harbin Medical University, Harbin, Heilongjiang, China; 2 Department of immunology, Harbin Medical University, Harbin, Heilongjiang, China; University of Turin, Italy

## Abstract

Accumulating evidence shows a tight link between inflammation and cancer. However, comprehensive identification of pivotal transcription factors (i.e., core TFs) mediating the dysregulated links remains challenging, mainly due to a lack of samples that can effectively reflect the connections between inflammation and tumorigenesis. Here, we constructed a series of TF-mediated regulatory networks from a large compendium of expression profiling of normal colonic tissues, inflammatory bowel diseases (IBDs) and colorectal cancer (CRC), which contains 1201 samples in total, and then proposed a network-based approach to characterize potential links bridging inflammation and cancer. For this purpose, we computed significantly dysregulated relationships between inflammation and their linked cancer networks, and then 24 core TFs with their dysregulated genes were identified. Collectively, our approach provides us with quite important insight into inflammation-associated tumorigenesis in colorectal cancer, which could also be applied to identify functionally dysregulated relationships mediating the links between other different disease phenotypes.

## Introduction

The close link between inflammation and cancer in the intestine has been appreciated for centuries based on clinical observations [1], [2]. Inflammatory bowel diseases (IBDs), which include ulcerative colitis (UC) and Crohn’s disease (CD), predispose patients to the development of colorectal cancer (CRC) [3], which is one of the most common and fatal cancers worldwide. Although, the ‘adenoma-carcinoma’ sequence has been long of central importance to studies on CRC, a shift in the focus to the sequence of ‘inflammation-dysplasia-carcinoma’ has been observed [4]. One possible explanation [5], [6] could be that inflammation, which expedites the acquisition of cancer hallmarks underlying the injured colonic tissues, could promote tumorigenic progression. However, interpretation of the tight links bridging inflammation and cancer in the intestine remains challenging.

High-throughput technologies have greatly promoted the production of vast amounts of multiple-layer biological data, for example gene expression microarray [7], [8]; these data have be extensively used to characterize the molecular differences between normal and malignant cells [9], [10], or molecular associations between distinct disease phenotypes [11], for example inflammation and cancer. These expression-based studies successfully identified individual genes involved in phenotypic characterization, whereas it is still difficult to infer any details of relationships between these molecules underlying oncogenesis. Therefore, it is reasonable to identify relationships altered or dysregulated at a pathway or network level.

Much of a cell’s response to the internal or external stimuli is governed by a global regulatory network mostly at the transcriptional level [12]. As one of the major regulators in mammal cellular context, transcription factors (TFs) significantly contribute to several pathological processes. Greten et al. [13] showed that the specified component of transcription factor *NF-κB* linked inflammation and tumorigenesis in UC-related CRC, using a knockout mouse model. A recent work [14] implicated transcription factor *STAT3* in cell survival and cell-cycle progression of colitis-associated tumorigenesis. However, no systematic studies of TFs involved in the link in the intestine have been reported. Translating genome-wide expression data into network knowledge is essential for further large-scale analysis, which requires computational tools, such as coexpression or information-theoretic associated approaches [15]. Most recently, gene networks are typically constructed from gene expression data through computational analysis. The first large-scale analysis of microarray coexpression aimed to increase the inference stability of gene functions [16]. Subsequently, Choi et al. [17] compared a tumor and normal coexpression network constructed from 13 distinct cancer phenotypes, and then identified differential coexpression relationships with functional alterations. In addition to studies on these altered relationships, associated pathways or subnetworks are also identified via integrated network-based approaches. In the case of glioblastoma (GBM), Cerami et al. [18] confirmed that functional GBM alterations tend to occur within specific modules, and therefore they tried to identify GBM-related core pathways using automated network analysis. Simultaneously, functional sub-networks in colorectal cancer were recognized by Nibbe et al. [19] using random walk algorithm. Undoubtedly, these methods are useful in identifying associated molecular mechanisms underlying individual disease. However, studies on disease phenotype in response to molecular perturbations [20] or on molecular associations between distinct disease phenotypes are still in their early stage. Abdollahi et al. [21] demonstrated that the switch from angiogenic balance to a pro-angiogenic phenotype is governed by global transcriptional circuitries in pancreatic cancer in response to key endogenous proteins, based on a regulatory network. Another study on distinct stages of hepatocarcinoma completed by He et al. [22] identified potential molecular processes by applying a network approach in combination with transcriptional regulation.

**Figure 1:**
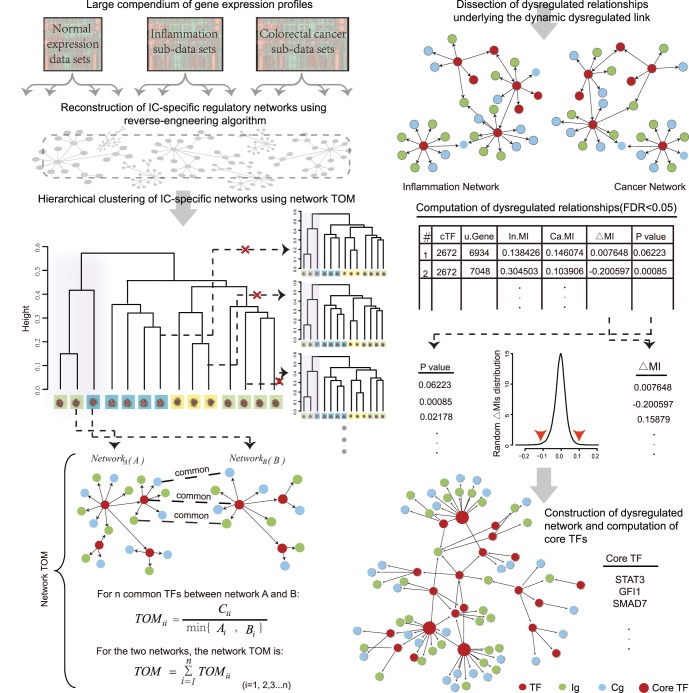
Workflow applied to identify core TFs. The procedure is mainly divided into four steps: 1) reconstruction of IC-specific regulatory networks from a large compendium of microarray data; 2) clustering of IC-specific regulatory networks using network TOM, followed by network perturbations; 3) construction of a dysregulated network with edges dysregulated between inflammation and their linked cancer network based on network comparisons; 4) identification of core TFs via dysregulated pattern analysis. TF, transcription factor; Ig, inflammation gene; Cg, colorectal cancer gene; MI, mutual information.

In this work, we adopt an integrated computational approach (Figure 1; see also Figure S1 for a flow chart of the computational steps) to reconstruct regulatory networks of normal, IBDs and CRC from a large compendium of gene expression profiling with distinct expression patterns of inflammation and cancer genes (referred to as IC-specific networks), using a reverse-engineering algorithm. A network-based clustering is then applied to characterize a potential clue linking IBDs and associated CRC networks, which assists us to distinguish inflammatory networks with tumorigenic potentials. Through network comparison analysis, dysregulated relationships are computed with significant gain or loss of mutual information between inflammation and cancer network, and then a dysregulated network is constructed. Based on dysregulated pattern analysis, we finally identify 24 pivotal TFs (i.e., core TFs), together with their dysregulated genes, as interesting candidates for biologists; this will surely extend and complement the current knowledge of inflammation-related tumorigenesis in colorectal cancer.

## Materials and Methods

### Data Sources

We collected 13 IBDs and CRC-related gene expression data sets from Gene Expression Omnibus (GEO) (Table S1). Their corresponding processed series matrix files were used as data input to re-construct gene interaction networks. Background correction and data normalization of each expression data set were already performed, separately. Probe sets mapped to none or multiple human Gene IDs were removed. Expression values were log2 transformed. For each data set, we extracted samples in conditions of UC, CD, CRC, and normal, which resulted in 22 expression data sets.

We obtained 231 inflammation-related genes from the Gene Ontology categories “inflammatory response” (GO:0006954) and “regulation of inflammatory response” (GO:0050727), which were then referred to as the inflammation gene set. The colorectal cancer gene set (cancer gene set), which included 196 genes, was manually generated from the Online Mendelian Inheritance in Man database (OMIM) by searching different key words (“colorectal cancer” OR “colorectal carcinoma” OR “colorectal neoplasm”). These two gene sets are then referred as IC gene sets. Besides, a TF set referring to 344 unique TFs was downloaded from TRANSFAC® Professional 11.4.

### Reconstructing IC-specific Regulatory Networks

First, the K-means clustering algorithm [23] was applied to IBD and CRC-related expression data sets based on the expression patterns of genes in both inflammation and cancer gene sets, identifying expression-homogeneous sample groups that were then referred to as IC-specific data sets. For each of the UC, CD, and CRC related data sets with more than 100 samples, we grouped samples into four clusters using K-means clustering. Those with less than 100 samples were grouped into two clusters. The sub-divided expression profiling or IC-specific expression data sets, including normal data sets not subjected to clustering treatment, were generated. Then, those with less than 20 samples were excluded from further study, in consideration of the precision of the approach used for the construction of regulatory networks [24]. The data sets not excluded were used for the reconstruction of IC-specific regulatory networks.

ARACNe (Algorithm for the Reconstruction of Accurate Cellular Networks) [25], which is based on an information-theoretic approach and data processing inequality (DPI) control, provides a way to infer regulatory networks directly from gene expression data. Interactions between TFs and genes were identified by computing mutual information (MI) estimated by the Gaussian kernel method with a specified *p-*value cutoff, which were then pruned by DPI analysis based on a tolerance parameter. Based on the TF set derived from TRANSFAC, we used the ARACNe program to reconstruct one regulatory network between TFs and all genes detected by the microarray screening from each IC-specific expression data set independently, with a *p-*value cutoff of 0.001 and a stringent cutoff on DPI tolerance of 0%. Then we extracted IC and TF genes from the constructed regulatory network, which are termed as IC-specific regulatory network.

### Clustering of IC-specific Regulatory Networks

A modified version of topological overlap measure (TOM) [26], [27] named network TOM was proposed to compute the similarity of regulatory properties for all common TFs between each two regulatory networks, when performing clustering of IC-specific networks. Given two regulatory networks, let 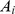 and 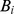 be the number of neighbors of common 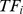 in 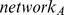 and 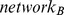, respectively. The number of common neighbors of 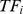 was represented by 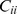, and then we could define the 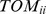 for any common 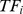 as below:
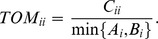



Finally, 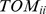 was summed into network 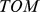 between the two networks and then divided by the maximum network TOM for all possible pairwise networks, which was then used as the measure of similarity between the two networks.

Next, network permutations were applied to those IC-specific networks and then new clustering results were generated via repeatedly removing one network out for all the networks used for network clustering and then two networks out, using the same similarity measure.

### Computing Dysregulated Relationships

We define a regulatory relationship between 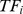 and 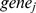 to be dysregulated between inflammation and cancer network, if and only if the MI difference of the relationship between IBD and CRC networks is statistically significant in comparison with random distribution.

Given a pair of IBD and CRC networks, we combined those adjacent neighbors of each common 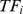 in the individual network and then computed the MI difference for each relationship of all common TFs. The MI difference between 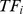 and 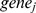 was computed as below:
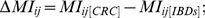
where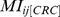 and 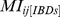 represent MI between 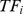 and 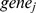 in the CRC and IBD networks, respectively. All MI values were computed from the ARACNe program. Finally, we generated all MI differences for all relationships of all common TFs between the two networks. To identify dysregulated relationships, permutation tests were done 100 times for both IBD and CRC expression data sets, resulting in 100 pairs of random networks computed from the corresponding pair of random data sets. We repeatedly computed the MI difference for each relationship using each pair of random IBD and CRC networks obtained above and then merged all MI differences into a random distribution. A false discovery rate (FDR) *p-*value <0.05 was used as significance cutoff. Those dysregulated relationships were visualized using Cytoscape software [28]. The largest connected component was extracted for further analysis.

### Identifying Core Transcription Factors

A TF is defined to be a pivotal regulator in the dysregulated network constructed out of dysregulated relationships between IBD and CRC networks, based on the degree distribution of the TF and the composition ratios of its directly connected inflammation and cancer genes. As for the composition ratios of each TF, we computed the ratio of the number of adjacent inflammation genes to its degree, and the ratio of the number of adjacent cancer genes to its degree.

## Results

### Reconstruction of IC-specific Normal, IBD, and CRC Regulatory Networks

We collected twelve gene expression data sets from the GEO database (Table S1). Out of these data, we extracted 22 sets of normal, UC, CD, and CRC expression data (normal: 7; UC: 6; CD: 3; CRC: 6) referring to 1201 samples in total. Firstly, to generate relatively homogeneous samples, we applied a K-means clustering algorithm to the 22 expression data sets based on the distinct expression patterns of 196 cancer and 231 inflammation genes. This resulted in 14 UC, 6 CD, and 16 CRC subsets, which are referred to as IC-specific. The other 7 normal data sets, which were not subjected to K-means clustering analysis, are believed homogenous and also included for further analysis. Finally, twenty-one IC-specific expression data sets were retained, including 5 UC, 2 CD, 11 CRC, and 3 normal, with at least 20 samples for each.

Then, we reconstructed 21 IC-specific regulatory networks from the corresponding IC-specific expression data sets using the ARACNe program with a P-value and a DPI tolerance as described in Materials and Methods. ARACNe, as a reverse-engineering algorithm, is widely used to reconstruct gene interaction networks in mammalian cellular context. As comparing with other algorithms in the same family, ARACNe algorithm is deemed good in performance when dealing with steady-state data (not time-series) and is still outstanding when few experiments are available, as compared with the number of genes [29], [30], [31]. The inferred networks contained TFs and all their potentially connected genes. To further explore those underlying pivotal factors mediating the dysregulated links, TFs and their directly connected IC genes were extracted, and then the maximal connected component for each IC-specific network was used for the following analysis (Figure S2).

**Figure 2:**
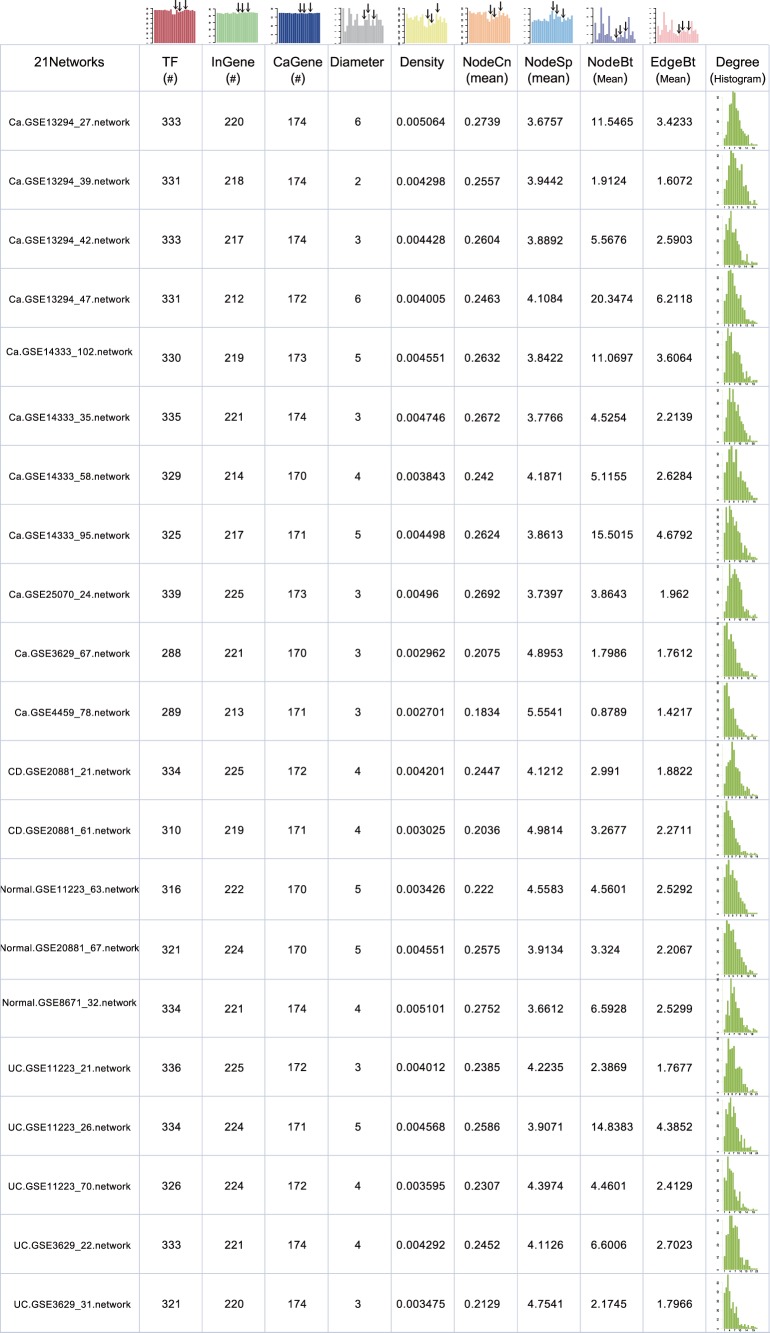
Network topological parameters of IC-specific regulatory networks. The respective numbers of TFs, inflammation and cancer genes are listed in the first three columns. Network diameter, network density, mean node closeness (only TFs), mean node shortest paths (only TFs), and mean betweenness of nodes (only TFs) and edges (all edges) are also included in the following six columns. For the first nine columns, each is accompanied with a histogram outside the first row, with the height of each bar indicating the number in each cell of the corresponding column. The degree distribution of only TFs in each network is provided as histograms in the last column. The three black down-arrows in each histogram classify all bars into four groups, representing cancer, CD, normal, and UC networks, respectively. InGene, inflammation gene; CaGene, cancer gene; #, number; NodeCn, node closeness; NodeSp, node shortest paths; NodeBt, node betweenness; EdgeBt, edge betweenness.

Figure 2 lists the network topological parameters of 21 IC-specific networks, including network diameter, network density, mean node closeness, mean node shortest paths, betweenness, and degree. Network constitutions, i.e. the number of TF, inflammation genes and cancer genes corresponding to each network are also provided. We observe that the nodes are constituted similarly among networks. The number of TFs, inflammation or cancer genes in each network shows small alterations, ranging from 288 to 339, 212 to 225, and 170 to 174, respectively. Moreover, the respective numbers of node constitutions for common TFs, inflammation or cancer genes between each two IC-specific networks are also parallel with each other (details in Figure S3). However, certain topological parameters show obvious discordances among normal, inflammation or cancer regulatory networks. For example, mean node (only TFs) and edge (all edges) betweenness show great differences with each other even among cancer network themselves. While mean node (only TFs) closeness and shortest paths show moderate differences when inflammation and cancer networks are compared.

### A Potential Clue Linking IBDs and Associated CRC

**Figure 3:**
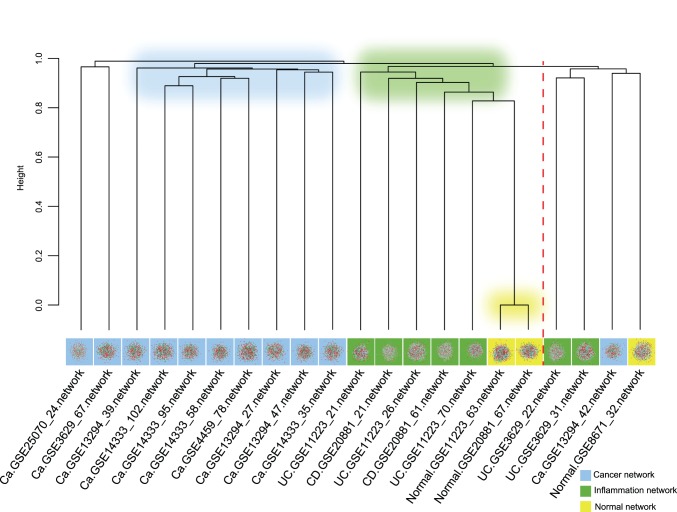
Clustering of IC-specific regulatory networks. Network TOM was used as the similarity measure of hierarchical clustering. Three representative branches are marked by different color shadow. Ca, cancer network; UC and CD, inflammation networks; Normal, normal network; Ca.GSE25070_24.network, the cancer network inferred from the GSE25070 data set with 24 samples after subjecting to K-means clustering algorithm.

Network TOM is used to assess the regulatory similarity of common TFs between each two different networks. We then used the network similarity measure to cluster 21 IC-specific regulatory networks. As indicated by the clustering result, normal, inflammation or cancer networks generally have the maximum similarity within their respective categories, such as the branch of eight tightly clustered cancer networks shown in Figure 3 (left of the red dashed line). Exactly, the nearby branch of five inflammation (including CD and UC), and two normal networks are also tightly clustered respectively. Those networks expected to generate the most closely associated regulatory patterns of TFs make up three major representative branches of normal, inflammation, and cancer (from right to left, marked by corresponding color shadow in Figure 3). Each branch means that these networks are much more parallel with each other in regulatory mechanisms (or regulatory patterns) than with those from other branches. Intuitionistically, two normal networks are clustered within the branch of inflammation networks, which we called the normal branch; their remarkably smaller distance is actually a good representation for the high performance of our proposed network TOM. Unsurprisingly, those tightly clustered inflammation or cancer networks generated relatively bigger distance are mainly due to disease heterogeneity [32].

**Figure 4:**
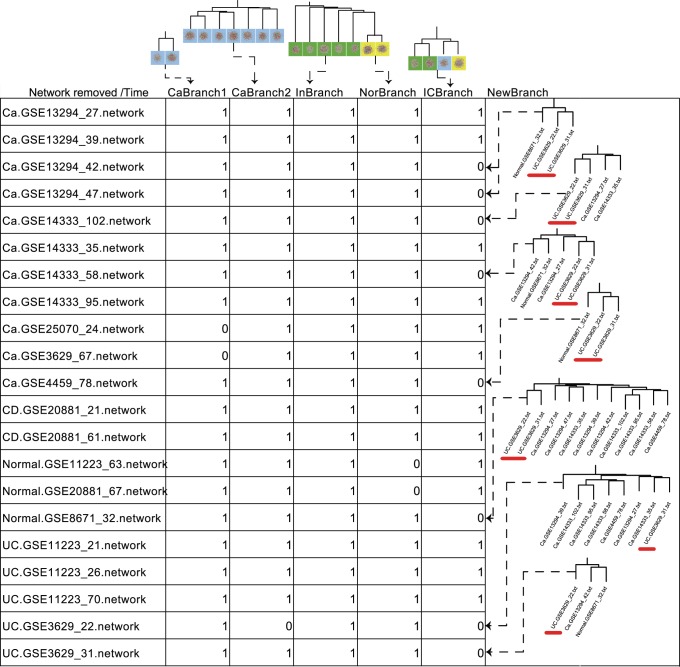
Frequently recurred ICBranch in network perturbation. We removed one network out and then generated new clustering, repeatedly, to examine whether those branches generated using all networks recurred or not. (Names of the removed networks are listed in the first column. The number 1 in each cell from column two to six means recurring, while the number 0 means not.). Branches classified as CaBranch1, CaBranch2, InBranch, NorBranch, ICBranch and NewBranch are manually extracted from the hierarchical clustering using all networks. CaBranch1, contains exactly the two networks of Ca.GSE25070_24.network and Ca.GSE3629_67.network. CaBranch2, contains cancer networks clustered closely with each other. InBranch, includes inflammation networks. NorBranch, contains exactly the two normal networks of Normal.GSE11223_63.network and Normal.GSE20881_67.network. ICBranch, contains exactly the four networks of UC.GSE3629_22.network, UC.GSE3629_31.network, Ca.GSE13294_42.network and Normal.GSE8671_32.network. NewBranch was shown in the right side, only and only if the ICBranch was missing. The UC networks in ICBranch are underlined by red line in NewBranch.

Interestingly, two UC networks, i.e., UC.GSE3629_22.network and UC.GSE3629_31.network, are clustered tightly together with one CRC network, i.e., Ca.GSE13294_42.network, in the rightmost branch (Figure 3, right of the red dashed line). In case of systematic noise, we tried to evaluate the recurrence of the exact branch by randomly removing one network out and then perform clustering on the remaining networks. Although perturbations of networks used for clustering could cause some alterations in final results, we are encouraged to see that the two UC networks are always clustered with the same CRC network (Figure 4). Consistently, the results for randomly removing two networks out also demonstrate that the exact branch recurs with the most frequency. Furthermore, as supported by the literature, patients with UC are more predisposed to colitis-associated cancers (CAC), such as CRC [33], and CRC is a major threat in long-standing UC patients [34], which partly supports the potential links implied by the branch. We thus reason that there exist some similar regulatory mechanisms between the inflammation and cancer regulatory network, which also suggest a potential functional link between UC and CRC. Meanwhile, one normal network (Normal.GSE8671_32.network) seemed also unexpectedly clustered within the same branch next to the cancer network. One possible explanation might be that the normal network derived from histological normal colonic tissues has already executed advanced molecular processes of inflammation and/or cancer beneath the normal presentation (Figure S4).

### Dysregulated Relationships between UC and their Linked CRC

Clustering of IC-specific regulatory networks based on network TOM assists us to delineate a potential clue linking UC and associated CRC, with the branch (Figure 3, right of red dashed line) providing us quite promising candidates. To interpret the dysregulated links, relationships with significant gain or loss of MI between the UC and CRC networks were identified. For the combination of UC.GSE3629_22.network and Ca.GSE13294_42.network, we firstly combined direct neighbors of common TFs, and then computed MI difference for each relationship. Secondly, 100 random IC-specific expression data sets for each of the two expression data sets, which were used for reconstructing corresponding regulatory networks, were generated. From each of the 100 pair random expression data sets, we repeatedly computed MI differences for those relationships computed above after reconstructing corresponding random regulatory networks using ARACNe program with default parameters, and then form a random distribution of MI differences. In comparison with random distribution, we could define the significance of each relationship by an FDR p-value <0.05. Those relationships with significant gain or loss of MI were considered as dysregulated. Then, we generated 3394 dysregulated relationships from the combination, while 2898 were generated from the other combination of UC.GSE3629_31.network and Ca.GSE13294_42.network. Finally, we extracted 1052 relationships, which were defined as dysregulated simultaneously in both combinations, to construct a dysregulated network.

The network, which contains 625 nodes with 285 TFs, and 200 inflammation and 162 cancer genes, is an objective representation of 1052 dysregulated relationships between TFs and IC genes. Key network topological parameters (Figure S5) demonstrate that it is a scale-free and small-world biological network.

### Core Transcription Factors Mediate the Dysregulated Links

**Figure 5:**
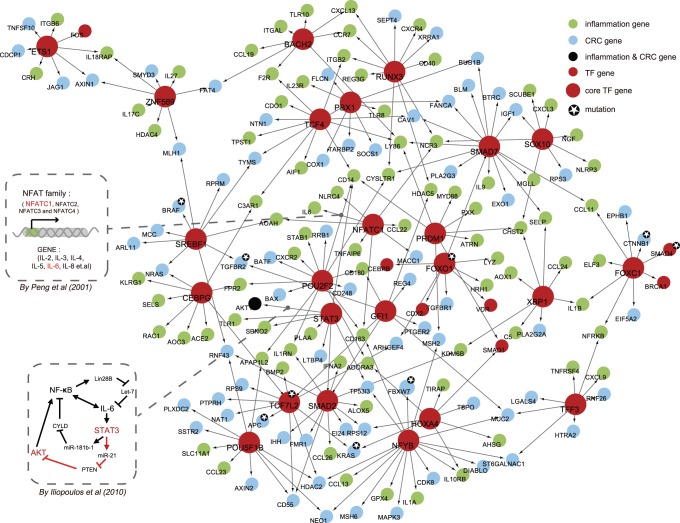
Core network constructed out of core TFs. The sub-network is constructed from 24 core TFs and their dysregulated IC genes. Several interactions have been confirmed by other researches. For example, the interactions between NFATC1 and IL6, and STAT3 and AKT1, which have been confirmed by biological experiments, are illustrated by the two inserted dashed rectangle and offered with detailed information on how it works. Core TFs are highlighted in red with bigger size. Genes with genetic variations offered by March et al. are marked with a small five-pointed star.

Transcription factor-mediated regulatory networks serve as a decision-making system within mammal cells [35]. Therefore, based on the dysregulated network constructed, we could identify core TFs functioning through regulating adjacent dysregulated genes bridging UC and associated CRC. After studying the degree distribution of all TFs in the network, we threshold a TF degree of no less than 8 to be topologically important. And then, we checked the composition ratio of adjacent inflammation and cancer genes of all TFs. As indicated, TFs could be categorized into three kinds: cancerogenic TFs with mostly adjacent cancer genes; inflammatory TFs with mostly adjacent inflammation genes; IC-specific TFs with both adjacent inflammation and cancer genes. In order to identify those potentially involved core TFs regulating not only inflammation genes, but also cancer genes, the composition ratios (including both the ratio of the number of adjacent inflammation, and the ratio of the number of adjacent cancer genes) were set as at least 0.1. In favor of our rules, 24 core TFs are generated based on these restrictions, considering both the degree constraint and the constituent ratio of their adjacent inflammation and cancer genes. Those prone to regulating only inflammation or cancer genes and with lower degree showing a relative minor influence on the whole network structure were not included. A dysregulated sub-network (core network) is then constructed out of 24 core TFs with their directly connected genes (Figure 5). The core TF list includes *STAT3*, *GFI1*, *NFATC1*, *TCF7L2*, *ETS1*, *CEBPG*, *XBP1*, *RUNX3*, *SMAD7*, *SMAD2*, *POU2F2*, *FOXC1*, *TCF4*, *PBX1*, *HOXA4*, *SOX10*, *SREBF1*, *NFYB*, *FOXO1*, *PRDM1*, *ZNF589*, *BACH2*, *POU5F1B*, and *TFF3*. Some core TFs, i.e. *TCF7L2* and *FOXO1* are important which are also targets for genetic mutations [36] (Figure 5).

Certain core TFs together with their dysregulated relationships are generally known, which highlights the importance in regulation of core TFs in bridging inflammation and cancer in the intestine. An example is *NFATC1* together with its dysregulated gene *IL6* [37]. Early activation of *IL6* is necessary for the malignant transformation of normal cells in mouse cell line model [38]. Most studies in T cells prove that the release of IL6 depends on the activation of *NFATC1* [39], which is also implicated in immune responses of multiple types of cells corresponding to intestinal injury. Another well-reported factor *STAT3* [40] is activated through phosphorylation in a manner that IL6 binds to its receptor. IL6 could induce the transcription of *STAT3*, and then accomplish its anti-apoptotic and pro-tumorigenic effects through *STAT3* and its downstream targets like *AKT1* [41]. *AKT1* [42], [43] is essential in regulating mammalian cell proliferation and survival. Moreover, the regulatory relationship between them has been already confirmed by Iliopoulos et al. [44]; this relationship is mediated by miR-21 and its target gene *PTEN*. *STAT3* can induce tumorigenicity of transformed cells and subsequent activation of *NF-κB* [45], which is another way of activating *IL6* [46].

## Discussion

Cancer research [47], [48] has generated a conceptual framework that is useful to understand the complex and dynamic alterations in cancer biology. The vast catalog of cancer phenotypes and genotypes is a full manifestation of six general hallmarks (or traits) enumerated by Hanahan et al., together with inflammation, which is recently known as ‘the seventh’ [49]. Although years of clinical and epidemiological researches have offered accumulated proofs on the strong association between inflammation and various cancer phenotypes, few studies have comprehensively evaluated the core transcription factors mediating the dysregulated links between IBDs and associated CRC.

The roles of inflammation in carcinogenesis are quite complex and are poorly understood, even though we ignore the direction of causation between inflammation and cancer. Therefore, on the basis of a limited number of cancer and inflammation samples, it is very difficult to delineate the mysterious and complex links between inflammation and cancer. Here, we integrated a large compendium of microarray expression profiles on the level of network, and separately extracted inflammation and cancer samples with similar transcriptional levels of inflammation and cancer related genes. These sample subsets enable us to depict complex and heterogeneous mechanisms of inflammation and cancer, and importantly, can help us capture potential mechanical links between inflammation and cancer.

To comprehensively characterize the mechanisms under different inflammation and cancer sample subsets, we constructed a series of inflammation and cancer regulatory networks independently using ARACNe algorithm, based on those sub-divided expression profiles each with at least 20 samples. The effects of such sample size on the performance of ARACNe have been studied and evaluated. For example, Altay et al. [24] compared ARACNe with other algorithms using sample size 20 and 200. Even though the sample size was relatively small, they claimed that the algorithm was still better in performance. Additionally, we also set a stringent threshold of the DPI parameter to prune edges in inferred networks. Furthermore, Such network-based strategy has been widely used to describe complex mechanisms underlying diverse disease phenotypes [50]. These networks allow us to find out whether there exist functional associations between some inflammation and cancer networks. A network-based clustering approach named network TOM was then proposed to find such a potential clue linking inflammation and cancer, via integrating several expression data sets with different platforms. Indeed, such network integration algorithm was minimally affected by the inter-platform difference [51], as suggested by the hierarchical clustering that, those networks were clearly grouped into three major branches (namely, cancer, inflammation and normal branch). Obvious structure similarity or regulatory similarity was observed among tightly clustered networks in each branch, especially the normal branch as expected although the networks were constructed using expression data from different platforms. It was undoubtedly that we could do much better using a large compendium of expression data sets with unified platform. Additionally, in consideration of the data heterogeneity not only from platform, several other researches have also introduced network integration in stead of directly assembling expression profiles to study the functional links among gene pairs [52], [53], [54]. Besides the three major successfully clustered branches of normal, inflammation and cancer, a special branch of tightly clustered inflammation and cancer networks was also identified. In order to confirm the robustness of the branch, we also performed permutation tests to construct new clustering results via repeatedly removing one network out. As indicated, the network clustering results generated in network permutation were similar with the clustering using all networks, which also suggested that the platform had minimal effects on the network integration approach. To delineate the links in the intestine, we computed relationships dysregulated with significant gain or loss of mutual information between inflammation and cancer networks, and then generated 24 core TFs together with their connected IC genes.

**Figure 6:**
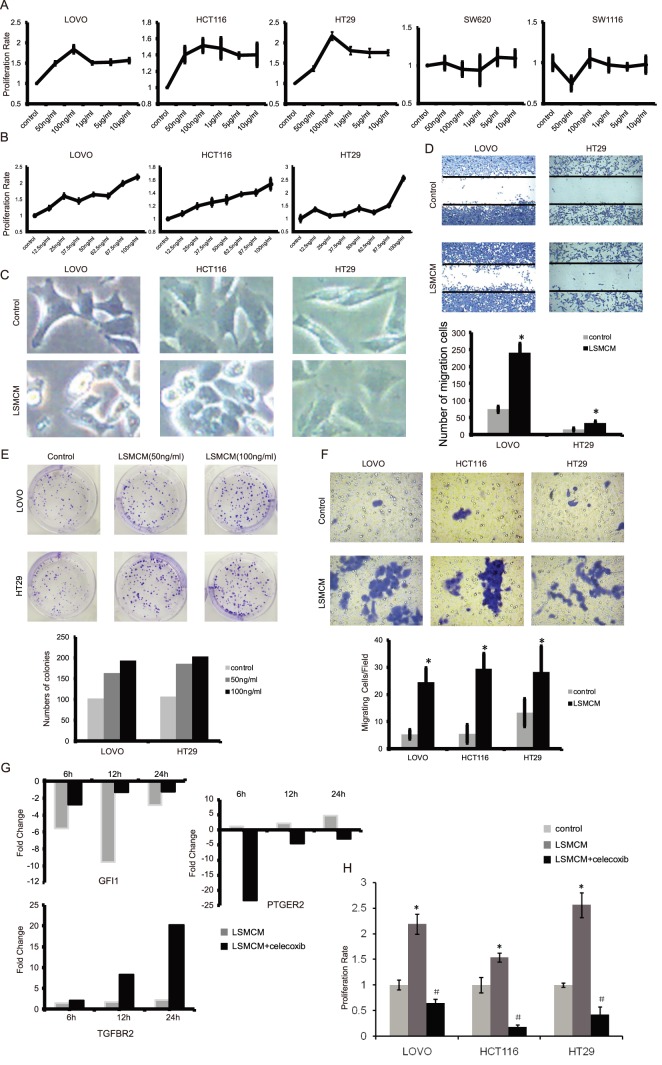
Verification of the emerging dysregulated relationships underlying the dysregulated links. (A) THP-1 cells were treated with different concentrations of LPS (0 ng/ml, 50 ng/ml, 100 ng/ml, 1 µg/ml, 5 µg/ml and 10 µg/ml, respectively), and then the human CRC cell lines were treated with these different supernatants. Cell proliferation rates were analyzed by MTT after 96 hours. (B) Positively responded cell line LOVO, HCT116, and HT29 were treated as in (A) with different concentrations of LPS (0 ng/ml, 12.5 ng/ml, 25 ng/ml, 37.5 ng/ml, 50 ng/ml, 62.5 ng/ml, 87.5 ng/ml and 100 ng/m). Proliferation rate of these cell lines raised with higher concentrations of LPS stimulation. (C) LOVO, HCT116, and HT29 cells were treated with LSMCM for 24 hours while the concentrations of LPS were 25 ng/ml for LOVO cells and 100 ng/ml the other two cell lines. Then cell morphology was examined with 400× enlargement. (D) Cells were treated as in (C) and the degree of cell migration was observed with 200× enlargement. (*p<0.05 vs. control). (E) Cells were treated with two kinds of LSMCM (THP-1 cells were stimulated with 50 ng/ml and 100 ng/ml LPS respectively), and the numbers of colonies were counted after 9 days. (*p<0.05 vs. control, ^#^p<0.05 vs. 50 ng/ml). (F) Cells were treated as (C) and the degree of cell invasion was observed with 400× enlargement.(*p<0.05 vs. control). (G) Cells were treated as (E) and total mRNA were collected at 6, 12 and 24 hours respectively. Celecoxib was used with LSMCM at the same time. Genes expression were detected by Real-time PCR. (H) Cells were treated as (A), and celecoxib was used with LSMCM at the same (*p<0.05 vs. control; ^#^p<0.05 vs. control).

In addition to computational analysis, we also designed a brief procedure to biologically examine those emerging dysregulated relationships between core TFs and IC genes (see Procedures S1). Using cell lines exposed to inflammatory stimuli, we observed that some selected core TFs and their connected IC genes were altered in expression at the mRNA level as examined by real-time PCR (Table S2). Meanwhile, we observed significantly malignant progressions such as enhanced proliferation (Figure 6A∼B), altered cell morphology (Figure 6C), and increased ability of cell migration (Figure 6D and 6F) and colony formation (Figure 6E). Moreover, we observed that expression of core TF *GFI1* and two of its dysregulated genes *TGFBR2* and *PTGER2* was time-dependent (Figure 6G), and furthermore suppressing *PTGER2* with celecoxib partly rescued the decrease of *GFI1* (Figure 6G ) and significantly inhibited cell proliferation rates (Figure 6H).

Additionally, we also tried to evaluate our approach by comparing it with differentially expressed genes identified between UC and CRC. Using data set GSE3629, which includes both UC and CRC expression data, 24 differential genes (Table S3) were identified using significance analysis of microarrays with a quite loose threshold of FDR<0.1. Compared with differential expression analysis, our proposed approach identifies certain known TFs, such as *STAT3*, whose dysregulated relationship with *AKT1* has been already validated by Iliopoulos et al. [44]. However, none of the core TFs or those well-known factors overlap with the differential gene list. One possible explanation could be that not all inflammatory samples undergo tumorigenic progression, and a mixture of inflammatory samples with and without cancerogenic traits would eliminate discordances in expression of certain pivotal factors. Moreover, the unsatisfactory performance of differential analysis also shows not only the complexity of the dysregulated links, but also a desperate need for an appropriate approach to study the underlying mechanisms mediated by core TFs between inflammation and cancer in the intestine.

## Supporting Information

Figure S1 Flow chart of the computational steps applied to compute core TFs. Computation process was provided, from the input of expression data sets, to the end of computation of core TFs. Details about network perturbation and corresponding sub-profiling permutation were included in the section of Materials and Methods in main text.
(EPS)

Figure S2 A full presentation of all 21 IC-specific regulatory networks. Each IC-specific regulatory network was reconstructed from corresponding IC-specific expression data set using ARACNe algorithm.
(EPS)

Figure S3 Heatmap of number of common TF, inflammation and cancer genes between each two IC-specific networks. Heatmap of number of common TFs (A), inflammation genes (B) and cancer genes (C) between each two IC-specific networks with columns and rows reordered based on corresponding means. Names of rows and columns correspond to all 21 IC-specific networks.
(EPS)

Figure S4 Relative expression of gene IL6 and STAT3 in three normal networks. We found that the expression level of IL6 (A) which could be an indicator of inflammation and function to regulate the survival and proliferation of intestinal epithelial cells was aberrantly lower in network GSE8671_32 than the other two normal networks. Similarly, STAT3 (B), another gene whose activation is generally dependent on IL6 binding to its acceptor and is mainly in charge of the protumorigenic and/or cytoprotective effects of IL6. Its expression level was significantly activated in network GSE8671_32 comparing to the other two normal networks. Thus, we inferred that the network might have already undergone certain local molecular processes of inflammation and/or cancer beneath the normal presentation. Height of each bar represents the mean of relative ranks of expression for the gene IL6 and STAT3 across samples in common genes between the three data sets. Error bar indicates 97.5% confidence interval. ***means significant comparisons and all p-values<0.001 (student’s t test) VS GSE8671_32.
(EPS)

Figure S5 Key topological features of the dysregulated network. (A) Shortest path length distribution. The average shortest path distance in the dysregulated network is 5.3597. (B) Degree distribution of the dysregulated network. The number of nodes with k neighbors, p(k), follows a power-law distribution: p(k)∼k^−γ^, γ = 1.996. Statistics on fitted line: R-square = 0.801.
(EPS)

Table S1 Thirteen IBD and CRC expression data sets downloaded from GEO database.^ a^represents the number of samples in status of normal, UC, CD, and CRC contained by each data set.
(XLS)

Table S2 Expression of some core TFs together with their dysregulated genes at the mRNA level in cell line model. Cells were treated with two kinds of LSMCM for 48 h (the supernatant of THP-1 cells which were treated with 50 ng/ml and 100 ng/ml LPS respectively). Gene expression showing at least 1.5 fold change was recognized as being significantly altered. A relationship is observed as dysregulated if no less than one node it connects is significantly altered in expression in at least one colon cell line as examined by real-time PCR. Consistent results from independent real-time PCR assays were obtained. ^#^undetected. Gene TGFBR2 and PTGER2 are shown in bold, which are used for further analysis (Figure 6G∼H in main text).
(XLS)

Table S3 Differential gene list using GSE3629 data set with FDR<0.1 using SAM.
(XLS)

Procedures S1 (1) Cancer cell lines and LPS-stimulated macrophage-conditioned medium. (2) MTT assays. (3) Boyden chamber migration assays. (4) Monolayer wound healing assays. (5) Colony formation assays. (6) Quantitative real-time RT-PCR analysis.
(DOC)
