# Supplementary material for: Systematic Identification of Core Transcription Factors Mediating Dysregulated Links Bridging Inflammatory Bowel Diseases and Colorectal Cancer
Source: PLoS One. 2013 Dec 26;8(12):e83495. doi: 10.1371/journal.pone.0083495 (PMC3873387; doi:10.1371/journal.pone.0083495)
Supplement: Procedures S1 — (1) Cancer cell lines and LPS-stimulated macrophage-conditioned medium. (2) MTT assays. (3) Boyden chamber migration assays. (4) Monolayer wound healing assays. (5) Colony formation assays. (6) Quantitative real-time RT-PCR analysis. (DOC) [file pone.0083495.s009.doc]

**Supplementary Experimental Procedures**

**Cancer cell lines and LPS-stimulated macrophage-conditioned medium**

Five human colorectal cancer cell lines, namely, SW620, SW1116, LOVO, HCT116, and HT29, and the human monocyte cell line THP-1 were originally obtained from the American Type Culture Collection. Among them, SW620, SW1116, LOVO, and HCT116 were maintained in Dulbecco’s modified eagle medium (DMEM), while THP-1 and HT29 cells were maintained in RPMI 1640 medium, all supplemented with 10% heat-inactivated fetal bovine serum (FCS) and 1% penicillin/streptomycin in a 37°C and 5% CO2 atmosphere. The medium and FCS were purchased from Hyclone (Shanghai, China).

To examine the effect of a mixture of inflammatory cytokines on the malignant potentials of CRC cells, the supernatant collected from bacterial LPS (Sigma, Shanghai, China) stimulated THP-1 cells for 6h, namely, LSMCM, was added to cell culture of the respective CRC cell lines. Varied concentrations of LPS, from 12.5 ng/ml to 10 μg/ml, were applied to obtain the optimized inflammatory condition on stimulating growth of the colorectal cancer cells.

**MTT assays**

The 96h MTT assay was used to measure the proliferation of SW620, SW1116, LOVO, HCT116, and HT29 cells under LSMCM treatment. Briefly, 1500 ~ 2000 cells were seeded per well of a 96-well plate and cultured with serum-free medium overnight. The next day, the cell cultural medium was replaced with/without varied LSMCM. At the end of the cell culture, the viability of the tested cells was determined by adding MTT (3-(4, 5-dimethylthiazolyl-2)-2, 5-diphenyltetrazoliumbromide, Sigma, Shanghai, China) to the cell culture. The formed dark crystals were dissolved by DMSO (Sigma, Shanghai, China), and the absorbance was measured at 490 nm with a micro-plate reader (Bio-Rad, Shanghai, China). Six replicate wells were used for each analysis, and at least three independent experiments were conducted.

**Boyden chamber migration assays**

The migration assay was performed in Boyden chamber units with transwell inserts (Costar, NY, USA), 6.5 mm in diameter with 8 µm pore size filters. Approximately 1*105 cells in 200 µl of LSMCM and serum-free medium (1:1) or 200 µl of serum-free medium were placed in the upper chamber, and 300 µl of the normal medium were placed in the lower chamber. The plates were incubated for 48 hours, then the cells were fixed in methanol for 15 minutes and stained with 0.05% crystal violet in PBS for 30 minutes. Cells on the upper side of the filters were removed with cotton-tipped swabs, and the filters were washed with PBS. Cells on the underside of the filters were examined and counted under a microscope.

**Monolayer wound healing assays**

LOVO, HCT116, and HT29 cells were seeded and grown with 10% FCS for 24h as a confluent monolayer in each well of six-well culture dishes. Before plating into the dish, two parallel lines were drawn at the underside of the well with a Sharpie marker. These lines served as fiducial marks for the wound areas to be analyzed. In preparation for making the wound, the growth medium was aspirated and replaced with PBS to prevent the destruction of cells at the edge. Consistent wounds of ~ 400μm in width were made perpendicular to the marker lines with a white P10 pipet tip. Culture medium without FCS was replaced with/without treatment with LSMCM. The wounds were examined using phase contrast microscopy. Images were taken at 24h, and cell migration situation was determined by counting the number of cells in the wound area.

**Colony formation assays**

To assess the clonogenic ability *in vitro*, LOVO and HT29 cells were plated in six-well plates at low density of 500 cells per well and cultivated for 24h. Cells were incubated with/without different LSMCM for up to 9 days at 37°C with 5% CO2, and the cell medium was refreshed every three days. At harvest, colonies were fixed with 2% methanol and stained with 0.5% crystal violet (Sigma, Shanghai, China) for 20 minutes at room temperature. The plates were then rinsed several times with distilled water and air-dried afterwards. A colony that contained more than 500 cells was counted. Each experiment was carried out in duplicate. Each set of experiments was done at least three times. Photos of the six-well plates at the end of cell culture were taken with a digital camera (Nikon, F500, Japan).

**Quantitative real-time RT-PCR analysis**

Total RNA was extracted from cultured LOVO, HCT116, and HT29 cells using Trizol-Reagent (Invitrogen Ltd., Shanghai, China), and cDNA was synthesized using High-Capacity cDNA Reverse Transcription Kits (Applied Biosystem, Foster City, CA). Gene expression analyses were performed using the Power SYBR Green PCR Master Mix (Roche, Japan). The PCR conditions for all genes were as follows: 95°C initial activation for 10 minutes followed by 40 cycles at 95°C for 15s and 60°C for 60s, and fluorescence determination at the melting temperature of the product for 20s using an ABI Step One (Applied Biosystem). The relative expression at the mRNA level was calculated by the comparative Ct method (ΔΔCT), and the values were normalized as fold changes (2^-ΔΔCT^) in relation to the expression of housekeeping gene β-actin.

**Statistical analysis**

Values are presented as mean±SD (standard deviation). One-way ANOVA was carried out for multiple comparisons, and two-tailed t-tests were used for single comparisons. A *p*-value <0.05 was considered statistically significant. All experiments were repeated at least three times.
